# Effectiveness of Individual Cognitive Stimulation on Cognition in Mild Alzheimer's Disease: A Multicenter RCT

**DOI:** 10.1111/psyg.70109

**Published:** 2025-10-26

**Authors:** Susana I. Justo‐Henriques, Raquel Lemos, Pardis Rahmatpour, Rosa C. G. Silva, Janessa O. Carvalho, Oscar Ribeiro

**Affiliations:** ^1^ Polytechnic Institute of Beja Beja Portugal; ^2^ Health Sciences Research Unit: Nursing (UICISA: E), Nursing School of Coimbra Coimbra Portugal; ^3^ Champalimaud Research & Clinical Centre, Champalimaud Foundation Lisbon Portugal; ^4^ ISPA ‐ Instituto Universitário de Ciências Psicológicas, Sociais e da Vida Lisbon Portugal; ^5^ RISE‐Health, Nursing School of Porto (ESEP) Porto Portugal; ^6^ Nursing School of Porto Porto Portugal; ^7^ Department of Psychology Bridgewater State University Bridgewater Massachusetts USA; ^8^ RISE‐Health, Department of Education and Psychology University of Aveiro Aveiro Portugal

**Keywords:** Alzheimer's disease, cognitive function, cognitive stimulation, executive function, memory, older adults

## Abstract

**Background:**

Alzheimer's Disease (AD) is characterised by impairments across several neurocognitive domains, including memory and executive function. The study explored the effectiveness of a 3‐month individual Cognitive Stimulation (iCS) program in older adults with mild AD.

**Methods:**

A multicenter randomised controlled trial was conducted with 62 Portuguese older adults with mild AD. Participants were randomly assigned to either iCS (*n* = 33; 53%) or treatment as usual (TAU, *n* = 29; 47%). Cognitive outcomes were assessed at baseline, post‐intervention, and 12‐week follow‐up using standardised tests for global cognition, memory and executive function.

**Results:**

The iCS group showed a significant improvement in memory and executive function compared to the TAU group. The analysis of subscales revealed significant improvements in encoding and semantic memory (Memory Alteration Test) and free delayed recall (Free and Cued Selective Reminding Test). Adherence and engagement with the intervention were high.

**Conclusions:**

A 3‐month iCS program showed preliminary benefits in specific cognitive domains (memory and executive function) in older adults with mild AD, warranting further research with larger samples and longer follow‐up.

**Trail Registration:** Clinicaltrials.gov ID: NCT05433493; Effect of Individual Cognitive Stimulation on Memory and Executive Function in Older Adults With Alzheimer's Disease.

## 1 Introduction

Alzheimer's disease (AD) is a genetic and sporadic neurodegenerative disease with a progressive decline in memory, executive function, language and other cognitive domains [1, 2]. In persons with mild AD, symptoms commonly include learning and memory problems, impaired judgement, mood swings, depression and restlessness [3].

With the increasing number of older adults worldwide, especially in Portugal, there has been an increase in the number of AD patients, which is predicted to reach 504 351 by 2050 [4]. The long‐term nature of AD care imposes a significant burden on families, prompting the development of non‐strategies to complement standard treatment [5].

One of the non‐pharmacological approaches is cognitive stimulation therapy (CST), which is known to be an effective treatment option for maintaining cognitive function and improving the quality of life. CST involves structured cognitive and social activities delivered in group or individual formats and can be combined with pharmacological treatment [6]. It is grounded in principles such as implicit learning, cognitive enhancement, and language stimulation, and maintenance of social participation through improved cognitive and social functioning [5].

Several studies support the effectiveness of the CST. Systematic reviews have demonstrated its positive effects on cognition, memory, orientation, and emotional well‐being in people with mild to moderate dementia [5, 7]. In Portugal, local evidence also reports significant benefits in cognition, communication, and behaviour following CST implementation Alvares‐Pereira et al. [8].

Building on group CST, individual cognitive stimulation (iCS) was developed for persons who prefer or require one‐on‐one intervention [9]. Studies conducted with the iCS approach show an increase in the quality of life [10], and memory performance [11], including both immediate and delayed recall [12].

However, findings are not universally consistent. Some trials have found no significant benefits from iCS [13, 14], and systematic reviews suggest that group CST may yield more robust outcomes Rao et al. [15].

Given the variability in previous findings, this study aimed to evaluate the effectiveness of a 3‐month individual cognitive stimulation program on global cognition, memory, and executive function in older adults with mild AD in Portugal. The main hypothesis is that participants receiving iCS would demonstrate greater cognitive improvements than those receiving treatment as usual.

## 2 Method

### 2.1 Study Design

This study analysed a subsample from a multicenter, single‐blind, randomised controlled trial on individual cognitive stimulation (iCS) for older adults with Alzheimer's disease. Participants were randomised (1:1) to iCS plus treatment as usual (TAU) or TAU alone. The iCS received two 45‐min sessions per week for 12 weeks. Assessments occurred at baseline (T0), post‐intervention (T1), and 12‐week follow‐up (T2). The study followed CONSORT guidelines, including the extension for nonpharmacologic treatments [16, 17].

### 2.2 Participants

A nationwide call invited 13 social care institutions to recruit older adults with probable AD. Of 167 initially screened, 142 were enrolled. This analysis focuses on a subsample with mild AD (*n* = 62; iCS = 33, TAU = 29).

Inclusion criteria included: DSM‐5‐TR diagnosis of probable AD confirmed in health records [18]; MMSE score between 21 and 24 [19, 20]; age ≥ 65; Portuguese as native language; communication skills intact; and use of social care services for ≥ 3 months. Exclusion criteria included severe sensory/physical limitations, acute illness, or recent changes in psychoactive medication. Medication use was recorded; randomization was assumed to balance pharmacological effects.

Exclusion criteria included impaired communication; severe sensory and physical limitations; acute illness; aggressive behaviour; and recent initiation or reintroduction of psychoactive or antipsychotic medication.

Eligible participants were enrolled and assessed at baseline. Each institution hosted both iCS and TAU groups. Participants were randomised (1:1) using non‐stratified permuted blocks (DatInf RandList) to ensure allocation concealment.

Group allocation was concealed from participants and therapists until the start. Trained clinical psychologists, blinded to allocation, conducted individual assessments at T0, T1 and T2 in a quiet setting. The therapists at each institution administered the iCS sessions. The iCS sessions were conducted by 15 therapists and/or other rehabilitation professionals (e.g., psychologists, social educators, gerontologists, occupational therapists) who received a 6‐h group training on the intervention protocol (including objectives and schedule for each session, materials, and recommendations about the intervention) by two of the principal investigators for standardisation. Among the therapists and/or rehabilitation professionals, 80% were women, had a bachelor's or master's degree, were approximately 35 years old, and had 9 years of professional experience (with about 5 years of experience with iCS therapy specifically).

The study was approved by the Ethics Committee of the Health Sciences Research Unit: Nursing of Nursing School of Coimbra (number P876_05_2022), and all ethical principles were carefully maintained. All participants provided written informed consent after being informed of their rights, including voluntary participation and withdrawal. Therapists monitored engagement throughout the study.

### 2.3 Intervention

The intervention lasted 12 weeks (March 6th to May 26th, 2023). Each 45‐min session included four phases: welcoming (5 min), orienting (10 min), main activity (25 min) and closure (5 min). All sessions were one‐to‐one and included several activities based on the principles of iCS. The difficulty level increased from session to session. The current iCS program is conveniently described elsewhere [21, 22, 23]. Such activities were based on the therapeutic tools of CS, *Roletas da Memória* [Memory Roulettes], which comprised Portuguese language, math, and daily living activities, and *Bingos Seniores* [Senior Bingos], which include fruit bingo, travel to the past bingo and sound bingo.

Therapists completed the adherence form and could contact the principal researchers if needed. The sessions took place at each institution, scheduled by the therapist in distraction‐free settings [24].

The TAU group followed usual routines (e.g., social activities, light exercise, self‐care). Some may have engaged in occasional CS tasks, but no structured iCS was provided.

### 2.4 Outcomes' Instruments

Validated instruments assessed global cognition, memory, and executive function at T0, T1 and T2, administered by trained, blinded evaluators. At T0, a structured questionnaire was administered to collect participants' sociodemographic data, including direct questions about age, gender, educational level, marital status, type of institution attended, social response attended for at least 3 months and clinical condition. During each iCS session, the behavioural status of the participants was also evaluated.

Cognitive functioning was assessed using the Portuguese version of the MMSE (Cronbach's alpha = 0.89) and the Alzheimer's Disease Assessment Scale—Cognitive Subscale (ADAS‐Cog; Cronbach's alpha = 0.55). MMSE assesses orientation, retention, attention, and calculation, delayed recall, language and visuoconstruction (range: 0–30; higher = better) [25, 26, 27]. ADAS‐Cog evaluates memory, orientation, language, praxis and constructive capacity. Scores range from 0 to 68 (higher = worse performance). Despite low internal consistency, it is widely used in AD clinical trials [28, 29, 30, 31, 32].

Memory function was measured using the Memory Alteration Test (MAT; Cronbach's alpha = 0.93) and the Free and Cued Selective Reminding Test (FCSRT; Cronbach's alpha = 0.92 for the immediate recall and 0.88 for the delayed recall). MAT evaluates orientation, encoding, semantic memory, and recall. Scores range 0–50 (higher = better). It is sensitive to mild cognitive decline [33, 34]. FCSRT is a memory test using semantic cues during learning and recall of 16 categorized words [35, 36, 37].

Executive function was assessed using the Frontal Assessment Battery (FAB; Cronbach's alpha = 0.83), which includes six subscales: conceptualization, mental flexibility, motor programming, sensitivity to interference, inhibition control, and environmental autonomy. Scores range from 0 to 18, with higher scores indicating better performance [38, 39].

### 2.5 Data Analysis

Chi‐square tests for categorical variables and *t*‐tests for continuous variables were performed to determine whether the groups were homogenous prior to treatment. No imputation of missed data was made; thus, only data from participants who completed the follow‐up assessment were analysed. The effects of iCS on outcomes were analysed using 2 × 3 repeated‐measures mixed ANOVAs, with Group Assignment as a between‐subjects factor and Time as a within‐subjects factor. The main effects of interest were the Group × Time (*G* × *T*) interactions. Pairwise comparisons between groups for T0, T1 and T2 and between times of assessment for iCS and TAU groups are also reported. The Greenhouse–Geisser correction was used when sphericity was violated, and the Bonferroni adjustment was adopted for multiple comparisons designed to locate the significant effects of a factor. Statistical analysis was performed using IBM SPSS Statistics, version 29.

## 3 Results

### 3.1 Sociodemographic and Clinical Characteristics

**FIGURE 1:**
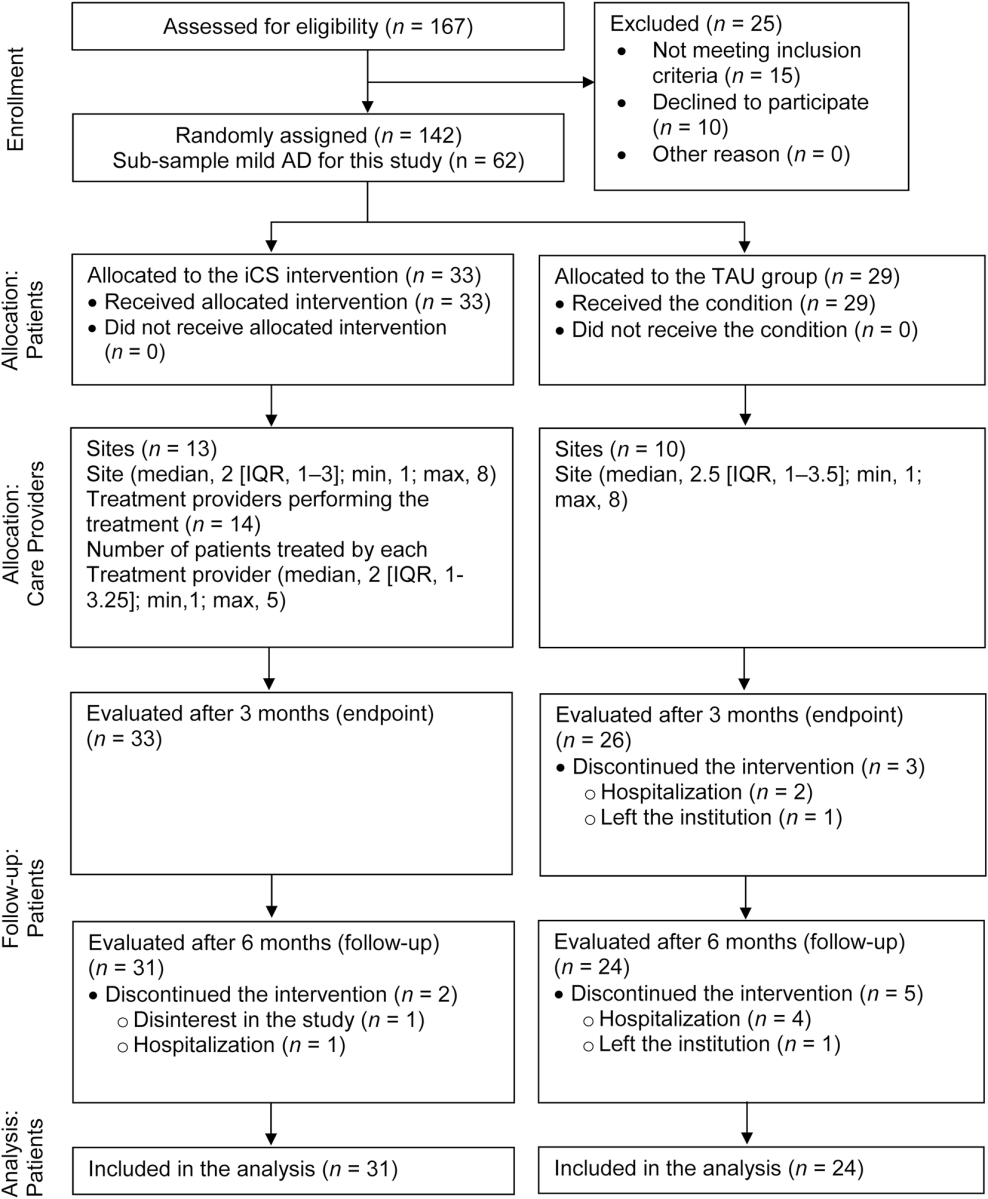
CONSORT (2017) [16, 17] diagram of participant flow through the study. Abbreviations: iCS, individual Cognitive Stimulation; IQR, interquartile range; TAU, treatment as usual.

Sixty‐two older adults with mild AD were included in this study: 33 in the experimental group and 29 in the control group. Figure 1 shows the participant flow through this trial. The participants' mean age was 82.7 (SD = 7.37) years old. Most were women (69.4%). Table 1 shows the participants' characteristics and assessment scores at baseline, as well as the results of between‐group comparisons. There were no significant differences between the two groups regarding demographic variables (all *X* ^2^ ≤ 2.54, *p* ≥ 0.168) and baseline mean scores for the outcomes (all *t* < 0.67, *p* > 0.446).

**TABLE 1:** Sociodemographic and clinical characteristics of the sample.

	*N* = 62	iCS group (*n* = 33)	TAU group (*n* = 29)	*t*, *χ* ^2^	*p*	*d*, *φ*, *U*
Age in years, mean (SD) [range]	82.7 (7.7) [65–98]	82.9 (7.9) [65–98]	82.4 (7.7) [65–94]	*t* = −0.27	0.792	*d* = 7.79
Gender (%)						
Male	19 (30.6)	13 (39.4)	6 (20.7)	*χ* ^2^ = 2.54	0.168	*φ* = 0.20
Female	43 (69.4)	20 (60.6)	23 (79.3)			
Educational level (%)						
1–4 years	40 (64.5)	19 (57.6)	21 (72.4)		0.263^a^	*φ* = 0.22
5–12 years	17 (27.4)	12 (36.4)	5 (17.2)			
Higher education	5 (8.1)	2 (6.1)	3 (10.3)			
Marital status (%)						
Married	18 (29)	10 (30.3)	8 (27.6)	*χ* ^2^ = 0.06	1.000	*φ* = 0.03
Widowed/divorced/single	44 (71)	23 (69.7)	21 (72.4)			
Type of institution attended (%)						
Home support service	7 (11.3)	2 (6.1)	5 (17.2)		0.373^a^	*φ* = 0.18
Day care	19 (30.6)	10 (30.3)	9 (31.0)			
Long‐term care centre	36 (58.1)	21 (63.6)	15 (51.7)			
Social response attended for at least 3 months (%)						
Yes	61 (98.4)	32 (97)	29 (100)		1.000^a^	*φ* = 0.12
No	1 (1.6)	1 (3)	0 (0)			
Antidepressants (%)						
Yes	33 (53.2)	18 (54.5)	15 (51.7)	*χ* ^2^ = 0.05	1.000	*φ* = 0.03
No	29 (46.8)	15 (45.5)	14 (48.3)			
Outcome						
MMSE score, mean (SD) [range]	22.35 (1.06) [21–24]	22.42 (1.12) [21–24]	22.28 (1.00) [21–24]		0.645	*U* = 510.00
ADAS‐Cog score, mean (SD) [range]	21.63 (6.36) [10–38]	22.21 (6.99) [10–38]	20.97 (5.60) [13–34]	*t* = −0.77	0.446	*d* = −0.20
MAT score, mean (SD) [range]	25.29 (8.15) [11–43]	25.27 (8.72) [11–43]	25.31 (7.61) [14–41]	*t* = 0.02	0.986	*d* = −0.01
FCSRT IR score, mean (SD) [range]	22.21 (11.40) [0–45]	23.12 (11.31) [0–44]	21.17 (11.61) [2–45]	*t* = −0.67	0.506	*d* = −0.17
FCSRT DR score, mean (SD) [range]	7.66 (4.74) [0–16]	7.79 (4.46) [0–16]	7.52 (5.11) [0–16]	*t* = −0.22	0.824	*d* = −0.06
FAB score, mean (SD) [range]	9.00 (2.75) [3–17]	8.85 (2.61) [3–14]	9.17 (2.93) [4–17]	*t* = 0.46	0.647	*d* = 0.12

*Note:* Results of between‐group comparisons.

Abbreviations: ADAS‐Cog = Alzheimer's Disease Assessment Scale‐Cognitive, FAB = Frontal Assessment Battery, FCSRT DR = Free and Cued Selective Reminding Test Delayed Recall, FCSRT IR = Free and Cued Selective Reminding Test Immediate Recall, iCS = individual Cognitive Stimulation, MMSE = Mini‐Mental State Examination, MAT = Memory Alteration Test, TAU = treatment as usual.

^a^ Fisher's exact test.

### 3.2 Effects of iCS at Endpoint and Follow‐Up

#### 3.2.1 Global Cognitive Functioning (MMSE and ADAS‐Cog)

ANOVA (Table 2) revealed no significant interaction *G* × *T* on the MMSE (*F*(_2,106_) = 0.65, *p* = 0.523, *η*_*p*_^2^ = 0.012), nor on the MMSE cognitive domains (Table S1).

**TABLE 2:** Results of repeated measures ANOVA.

	iCS (*n* = 31)			TAU (*n* = 24)			Group × time				Pairwise comparisons								
	T0 Mean (SD)	T1 Mean (SD)	T2 Mean (SD)	T0 Mean (SD)	T1 Mean (SD)	T2 Mean (SD)	df	*F*	*p*	*η*_*p*_^2^	iCS			TAU			iCS vs. TAU		
											T0 vs. T1	T0 vs. T2	T1 vs. T2	T0 vs. T1	T0 vs. T2	T1 vs. T2	T0	T1	T2
MMSE	22.52 (1.09)	23.29 (2.80)	23.42 (3.25)	22.33 (1.01)	22.63 (2.10)	22.50 (2.50)	2, 106	0.65	0.523	0.012									
ADAS‐COG^a^	22.29 (7.21)	18.39 (9.04)	19.42 (10.95)	21.17 (5.84)	21.08 (5.82)	21.04 (8.45)	1.43, 75.61	2.63	0.095	0.047									
MAT^a^	25.58 (8.87)	28.84 (10.75)	29.77 (10.82)	24.83 (7.29)	25.08 (7.26)	24.25 (7.67)	1.77, 93.56	5.31	**0.** **009**	0.091	**< 0.001**	**< 0.001**	1.00	1.00	1.00	1.00	0.740	0.147	**0.** **039**
FCSRT IR^a^	23.39 (11.62)	28.65 (12.58)	30.26 (13.53)	20.17 (11.84)	23.50 (11.54)	24.50 (11.20)	1.66, 87.82	1.24	0.288	0.023									
FCSRT DR^a^	7.81 (4.56)	9.45 (5.52)	9.65 (5.61)	7.00 (5.14)	7.13 (4.95)	7.58 (4.46)	1.62, 85.80	1.74	0.188	0.032									
FAB	8.90 (2.69)	10.26 (3.24)	9.94 (3.44)	9.33 (3.00)	8.63 (3.10)	8.71 (3.53)	2, 106	5.86	**0.** **004**	0.100	**0.** **008**	0.096	1.00	0.452	0.737	1.00	0.578	0.064	0.201

*Note:* Results of pairwise comparisons (Bonferroni correction) for T0, T1 and T2 assessments (iCS vs. TAU). Results of pairwise comparisons (Bonferroni correction) for iCS and TAU groups (T0 vs. T1, T0 vs. T2 and T1 vs. T2). The information in bold is statistically significant at an alpha level of 5%.

Abbreviations: ADAS‐COG = Alzheimer's Disease Assessment Scale‐Cognitive, FAB = Frontal Assessment Battery, FCSRT DR = Free and Cued Selective Reminding Test Delayed Recall, FCSRT IR = Free and Cued Selective Reminding Test Immediate Recall, iCS = individual Cognitive Stimulation, MMSE = Mini‐Mental State Examination, MAT = Memory Alteration Test, T0 = baseline assessment, T1 = post‐intervention assessment, T2 = 12‐week follow‐up assessment, TAU = treatment as usual.

^a^ Greenhouse–Geisser correction.

No significant *G* × *T* interaction was obtained on the ADAS‐Cog, (*F*(_1.43,75.61_) = 2.63, *p* = 0.095, *η*_*p*_^2^ = 0.047). Analysis of ADAS‐Cog components revealed a trend toward significant improvement in word recall (*p* = 0.046, *η*_*p*_^2^ = 0.057; pairwise comparisons for iCS group T0 vs. T1, *p* = 0.002 and T0 vs. T2, *p* = 0.017; Table S2).

#### 3.2.2 Memory Function (MAT and FCSRT)

A significant *G* × *T* interaction was found on the MAT (*F*(_1.77,93.56_) = 5.31, *p* = 0.009, *η*_*p*_^2^ = 0.091). Multiple comparisons showed that the iCS group significantly improved MAT scores from T0 to T1 (*p* < 0.001) and T2 (*p* < 0.001), while the TAU group maintained their scores across trials. iCS and control groups differed significantly at T2 (*p* = 0.039). Analysis of MAT subscales revealed significant improvements in encoding (*p* = 0.038, *η*_*p*_^2^ = 0.060; pairwise comparisons for iCS T0 vs. T1, *p* = 0.007 and T0 vs. T2, *p* = 0.013) and semantic memory (*p* = 0.006, *η*_*p*_^2^ = 0.093; pairwise comparisons for iCS group T0 vs. T1, *p* < 0.001 and T0 vs. T2, *p* < 0.001; iCS group vs. TAU group at T1, *p* = 0.048 and at T2, *p* = 0.006; Table S3).

For the FCSRT, the following measures were chosen for analysis: Total Immediate Recall—the sum of free and cued recall in the three trials (IR), and Total Delayed Recall—the sum of free and cued recall in the delayed trial (DR). ANOVA (Table 2) did not show a significant *G* × *T* interaction for IR (*F*(_1.66,87.82_) = 1.24, *p* = 0.288, *η*_*p*_^2^ = 0.023) and DR (*F*(_1.62,85.80_) = 1.74, *p* = 0.188, *η*_*p*_^2^ = 0.032). Analysis of FCSRT subscales revealed significant improvements in DR free (*p* = 0.013, *η*_*p*_^2^ = 0.084; pairwise comparisons for iCS, T0 vs. T1, *p* < 0.001 and T0 vs. T2, *p* < 0.001; iCS group vs. TAU group at T1, *p* = 0.015; Table S4).

#### 3.2.3 Executive Function (FAB)

A significant *G* × *T* interaction was obtained on the FAB scores (*F*(_2,106_) = 5.86, *p* = 0.004, *η*_*p*_^2^ = 0.100). Multiple comparisons showed that the iCS group significantly improved FAB scores from T0 to T1 (*p* = 0.008), while the TAU group maintained their scores across trials. The analysis of FAB subscales revealed a trend toward significant improvement in environmental autonomy (*p* = 0.050, *η*_*p*_^2^ = 0.058; iCS group vs. TAU group at T1, *p* = 0.017; Table S5).

### 3.3 Adherence to Intervention

Adherence to iCS sessions was high (Table 3). Mean attendance of participants was 23.12 sessions, with 97% attending more than 20 sessions; 63.6% attended all sessions. Twenty‐nine scheduled sessions were not held. The reasons for non‐attendance were: hospitalisation due to physical illness or a physical acute illness (62.1%), refusal to participate (20.7%), medical appointments (6.9%), family death (6.9%) and unjustified absence (3.4%).

**TABLE 3:** Attendance statistics for the iCS sessions.

Attendance	*n* = 33
Sessions attended	
Mean (SD) [range]	23.12 (1.87) [14–24]
Median [IQR]	24.00 [22.5–24]
Number of sessions attended (%)	
From 0 to 5	0 (0)
From 6 to 10	0 (0)
From 11 to 15	1 (3)
From 16 to 20	0 (0)
From 21 to 24	32 (97)

Abbreviation: iCS = individual Cognitive Stimulation.

### 3.4 Degree of Participation During the Intervention

After analysing the individual records of each session, we were able to obtain data regarding the level of collaboration of participants, which was operationalised by active participation in the iCS activities. Participation was very high. Of the 763 iCS sessions, the participants completed 727 (95.3%) of the activities presented. Participants appeared minimally engaged in 30 (3.9%) sessions and not engaged in only 6 (0.8%) sessions. In these cases, according to the qualitative judgement provided by the therapists at the end of each session, drowsiness or minimal attention and concentration prevented meaningful participation.

## 4 Discussion

This study presents the results from a 12‐week randomised controlled trial (RCT) on iCS for older adults with probable AD. Due to heterogeneity in baseline cognitive status (MMSE variation up to 14 points), the current analysis focuses on a subsample with mild AD (MMSE 21–24). The findings partially replicated those of the full RCT: [11] no significant effects were observed on global cognition, while small to moderate effects emerged on memory and medium effects on executive functioning.

While global cognition (MMSE, ADAS‐Cog) showed no significant changes, subscale analysis revealed improvements—especially in word recall, which was statistically significant (small effect size). A 3‐point change on ADAS‐Cog is considered clinically relevant in the literature [40]; differences exceeded the average (2.42) reported in a recent meta‐analysis by Woods et al. [41], though MMSE differences were below its pooled mean (1.99). It should be noted that the ADAS‐Cog is generally more sensitive and less influenced by education or language than the MMSE [42].

As for memory and executive functioning, there was a significant *G* × *T* interaction, with medium effect sizes. MAT subscales revealed significant gains in encoding and semantic memory, and minor changes were seen in temporal orientation, free recall, and cued recall. FCSRT subscales also revealed significant improvements in free delayed recall, with a medium effect size.

To date, most studies examining the cognitive domains that benefit the most from cognitive stimulation have focused on autobiographical memory, whereas only a few have explored other domains, such as semantic memory [43, 44, 45, 46]. In this study, improvements in semantic memory on the MAT are consistent with previous findings in autobiographical memory. In the FAB subscales, mental flexibility did not change, whereas all the other items improved in the intervention group; however, only in environmental autonomy was there a trend toward significant improvement, with a small effect size. Similar marginal effects on executive function were reported by López et al. [47]; Buschert et al. [48] additionally found a marginal significant effect on executive functions. In contrast, Kirk et al. [44] found no significant effects. Participant adherence was notably high: 97% attended 21–24 sessions (mean = 23.1/24), indicating strong engagement and satisfaction. Personalised, culturally adapted programs delivered by trained professionals can enhance engagement and adherence [49].

This study has several limitations, including a modest sample size, the absence of biomarker‐based confirmation of diagnosis, and a relatively short follow‐up period. Although shorter than the 6‐month follow‐up in Orrell et al. [50], our 12‐week follow‐up period corresponds to the interim assessment timepoint used in that trial, supporting partial comparability. These limitations, however, reflect the real‐world feasibility of conducting multicenter clinical trials without external funding. Importantly, in Portugal and in many clinical contexts, biomarker confirmation is not routinely performed prior to cognitive interventions; clinical diagnosis remains the standard of care, which enhances the ecological validity of our findings. However, this approach may allow for diagnostic misclassification, including the possible inclusion of cases such as LATE, tauopathies, or mixed dementia. Although no significant improvements were detected on global cognitive measures such as the MMSE and ADAS‐Cog, it should be noted that these instruments are not highly sensitive to short‐term cognitive changes in mild AD. Stability on these measures may itself be clinically meaningful in the context of a progressive neurodegenerative disease, while the observed gains in semantic memory and executive function highlight the potential of iCS to support domains that underpin communication, planning, and the execution of daily tasks—core components of functional independence and quality of life. As previous studies indicate, cognitive stimulation effects tend to diminish once sessions stop [42, 50]. Thus, future research should explore maintenance or longer‐duration protocols, alongside larger samples and biomarker‐based diagnosis, to better establish the clinical impact of iCS. In summary, while iCS did not produce significant changes in global cognition, it showed preliminary benefits in semantic memory and executive function. These findings should be interpreted with caution, but provide preliminary evidence that iCS may help maintain or enhance certain domain‐specific cognitive functions in mild AD. Further studies with larger samples and biomarker‐based diagnosis are warranted, as well as research on maintenance or longer‐duration protocols to better understand the sustainability of effects.

## Ethics Statement

The Health Sciences Research Unit: Nursing, Nursing School of Coimbra (Portugal) (Code number: P876_05_2022).

## Consent

All participants provided written informed consent after being informed of their rights, including voluntary participation and withdrawal.

## Conflicts of Interest

The authors declare no conflicts of interest.

## Supporting information


**Table S1:** MMSE cognitive domains. Results of repeated measures ANOVA.
**Table S2:** ADAS‐Cog components. Results of repeated measures ANOVA.
**Table S3:** MAT subtests. Results of repeated measures ANOVA.
**Table S4:** FCSRT subtests. Results of repeated measures ANOVA.
**Table S5:** FAB subtests. Results of repeated measures ANOVA.

## Data Availability

The data that support the findings of this study are available from the corresponding author upon reasonable request.
