# Supplementary material for: Effectiveness of Individual Cognitive Stimulation on Cognition in Mild Alzheimer's Disease: A Multicenter RCT
Source: Psychogeriatrics. 2025 Oct 26;25(6):e70109. doi: 10.1111/psyg.70109 (PMC12554431; doi:10.1111/psyg.70109)
Supplement: Supplementary file 1 — Table S1: MMSE cognitive domains. Results of repeated measures ANOVA. Table S2: ADAS‐Cog components. Results of repeated measures ANOVA. Table S3: MAT subtests. Results of repeated measures ANOVA. Table S4: FCSRT subtests. Results of repeated measures ANOVA. Table S5: FAB subtests. Results of repeated measures ANOVA. [file PSYG-25-0-s001.pdf]

Effectiveness of Individual Cognitive Stimulation on Cognition in Mild Alzheimer's Disease: A Multicenter RCT

Justo-Henriques et al., 2025; Article DOI: 10.1111/psyg.70109

SUPPLEMENTARY INFORMATION

Table S1. MMSE cognitive domains. Results of repeated measures ANOVA.

	**iCS (*n* = 31)**			**TAU (*n* = 24)**			**Group × time**			
	**T0 Mean (SD)**	**T1 Mean (SD)**	**T2 Mean (SD)**	**T0 Mean (SD)**	**T1 Mean (SD)**	**T2 Mean (SD)**	***df***	***F***	***p***	η_p^2
MMSE orientation	7.39 (1.36)	7.45 (2.22)	7.58 (2.03)	7.04 (1.33)	7.37 (1.28)	7.21 (1.32)	2, 106	0.39	0.681	0.007
MMSE retention[a]	2.97 (0.18)	3.00 (0.00)	2.97 (0.18)	2.92 (0.28)	2.92 (0.28)	2.88 (0.34)	1.80, 95.21	0.13	0.858	0.002
MMSE attention and calculation	3.58 (1.29)	3.81 (1.49)	3.74 (1.48)	3.75 (1.07)	3.42 (1.41)	3.58 (1.35)	2, 106	1.76	0.178	0.032
MMSE delayed recall	1.48 (0.96)	1.58 (1.09)	1.71 (1.13)	1.04 (0.86)	1.46 (0.88)	1.25 (0.79)	2, 106	1.35	0.265	0.025
MMSE language	6.74 (0.82)	6.94 (0.81)	7.13 (0.89)	7.21 (0.98)	7.08 (1.10)	7.25 (0.94)	2, 106	1.53	0.222	0.028
MMSE visuoconstruction	0.35 (0.49)	0.35 (0.49)	0.29 (0.46)	0.38 (0.50)	0.37 (0.50)	0.33 (0.48)	2, 106	0.02	0.978	0.000

Abbreviations*:* iCS = individual Cognitive Stimulation; MMSE = Mini-Mental State Examination; TAU = Treatment as usual; T0 = Baseline assessment; T1 = post-intervention assessment; T2 = 12-week follow-up assessment.

[a] Greenhouse–Geisser correction.

Effectiveness of Individual Cognitive Stimulation on Cognition in Mild Alzheimer's Disease: A Multicenter RCT

Justo-Henriques et al., 2025; Article DOI: 10.1111/psyg.70109

SUPPLEMENTARY INFORMATION

Table S2. ADAS-Cog components. Results of repeated measures ANOVA.

	iCS (*n* =31)			TAU (*n* = 24)			Group × time				Pairwise comparisons								
											iCS			TAU			iCS vs. TAU		
	T0 Mean (SD)	T1 Mean (SD)	T2 Mean (SD)	T0 Mean (SD)	T1 Mean (SD)	T2 Mean (SD)	*df*	*F*	*p*	η_p^2	T0 vs. T1	T0 vs. T2	T1 vs. T2	T0 vs. T1	T0 vs. T2	T1 vs. T2	T0	T1	T2
ADAS-Cog word recall	6.06 (1.50)	5.35 (1.94)	5.48 (2.01)	5.71 (1.43)	5.75 (1.15)	5.67 (1.49)	2, 106	3.18	**0.046**	0.057	**0.002**	**0.017**	1.00	1.00	1.00	1.00	0.377	0.381	0.711
ADAS-Cog naming objects and fingers	0.81 (0.91)	0.71 (0.78)	0.55 (0.85)	0.58 (0.50)	0.75 (0.85)	0.63 (0.58)	2, 106	0.84	0.436	0.016									
ADAS-Cog commands[a]	0.94 (0.85)	0.68 (1.08)	0.87 (1.09)	1.25 (1.26)	1.08 (0.97)	1.04 (1.12)	1.66, 87.76	0.35	0.668	0.006									
ADAS-Cog constructional praxis	2.00 (0.97)	1.61 (1.26)	1.97 (1.38)	1.62 (1.21)	1.67 (1.40)	1.88 (1.33)	2, 106	1.06	0.350	0.020									
ADAS-Cog ideational praxis[a]	1.13 (1.43)	0.87 (1.31)	0.87 (1.18)	0.96 (1.00)	1.04 (1.04)	0.83 (1.01)	1.59, 84.09	0.52	0.554	0.010									
ADAS-Cog orientation	2.81 (2.02)	2.39 (2.42)	2.65 (2.63)	2.54 (1.79)	2.46 (1.74)	2.33 (1.43)	2, 106	0.52	0.597	0.010									
ADAS-Cog word recognition[a]	5.39 (2.72)	4.35 (2.95)	4.65 (3.13)	5.00 (2.57)	5.38 (2.46)	5.29 (2.39)	1.45, 76.68	3.19	0.062	0.057									
ADAS-Cog remembering test instructions	1.16 (1.27)	0.87 (1.28)	0.81 (1.42)	1.13 (1.03)	0.83 (1.05)	1.00 (1.41)	2, 106	0.20	0.820	0.004									
ADAS-Cog language[a]	0.58 (0.96)	0.42 (0.76)	0.42 (0.76)	0.71 (0.91)	0.62 (0.82)	0.79 (1.10)	1.73, 91.84	0.75	0.464	0.014									
ADAS-Cog word finding difficulty[a]	0.65 (0.88)	0.65 (1.05)	0.61 (0.92)	0.87 (0.68)	0.75 (0.90)	0.75 (0.79)	1.68, 88.85	0.19	0.792	0.004									
ADAS-Cog comprehension of spoken language	0.77 (0.76)	0.48 (0.85)	0.55 (0.85)	0.79 (0.78)	0.75 (0.74)	0.83 (1.05)	2, 106	0.79	0.457	0.015									

Note: Results of pairwise comparisons (Bonferroni correction) for T0, T1, and T2 assessments (iCS vs TAU). Results of pairwise comparisons (Bonferroni correction) for iCS and TAU groups (T0 vs T1, T0 vs T2, and T1 vs T2). The information in bold is statistically significant at an alpha level of 5%.

Abbreviations: ADAS-Cog = Alzheimer's Disease Assessment Scale-Cognitive; iCS = individual Cognitive Stimulation; TAU = Treatment as usual; T0 = Baseline assessment; T1 = post-intervention assessment; T2 = 12-week follow-up assessment.

[a] Greenhouse–Geisser correction.

Effectiveness of Individual Cognitive Stimulation on Cognition in Mild Alzheimer's Disease: A Multicenter RCT

Justo-Henriques et al., 2025; Article DOI: 10.1111/psyg.70109

SUPPLEMENTARY INFORMATION

Table S3. MAT subtests. Results of repeated measures ANOVA.

	**iCS (n = 31)**			**TAU (n = 24)**			**Group × time**				**Pairwise comparisons**								
											**iCS**			**TAU**			**iCS vs. TAU**		
	**T0 Mean (SD)**	**T1 Mean (SD)**	**T2 Mean (SD)**	**T0 Mean (SD)**	**T1 Mean (SD)**	**T2 Mean (SD)**	***df***	***F***	***p***	η_p^2	**T0 vs. T1**	**T0 vs. T2**	**T1 vs. T2**	**T0 vs. T1**	**T0 vs. T2**	**T1 vs. T2**	**T0**	**T1**	**T2**
MAT encoding	6.10 (2.59)	7.23 (2.65)	7.10 (2.68)	6.79 (2.19)	7.00 (2.09)	6.50 (2.23)	2, 106	3.37	**0.038**	0.060	**0.007**	**0.013**	1.00	1.00	1.00	0.552	0.296	0.733	0.382
MAT temporal orientation	2.90 (1.47)	3.26 (1.79)	3.42 (1.84)	3.04 (1.43)	3.50 (1.10)	3.25 (1.48)	2, 106	0.66	0.520	0.012									
MAT semantic memory	8.74 (2.65)	10.26 (2.79)	10.65 (2.86)	8.58 (2.80)	8.79 (2.48)	8.29 (3.22)	2, 106	5.44	**0.006**	0.093	**< 0.001**	**< 0.001**	1.00	1.00	1.00	1.00	0.831	**0.048**	**0.006**
MAT free recall[a]	2.52 (2.05)	2.71 (2.41)	2.90 (2.72)	2.29 (2.42)	1.63 (2.02)	1.71 (1.71)	1.76, 93.27	2.14	0.130	0.039									
MAT cued recall	5.32 (3.18)	5.39 (3.26)	5.71 (3.22)	4.12 (2.83)	4.17 (3.17)	4.50 (2.70)	2, 106	0.00	0.999	0.000									

Note: Results of pairwise comparisons (Bonferroni correction) for T0, T1, and T2 assessments (iCS vs TAU). Results of pairwise comparisons (Bonferroni correction) for iCS and TAU groups (T0 vs T1, T0 vs T2, and T1 vs T2). The information in bold is statistically significant at an alpha level of 5%.

Abbreviations: iCS = individual Cognitive Stimulation; MAT = Memory Alteration Test; TAU = Treatment as usual; T0 = Baseline assessment; T1 = post-intervention assessment; T2 = 12-week follow-up assessment.

[a] Greenhouse–Geisser correction.

SUPPLEMENTARY INFORMATION

Table S4. FCSRT subtests. Results of repeated measures ANOVA.

	**iCS (*n* = 31)**			**TAU (*n* = 24)**			**Group × time**				**Pairwise comparisons**								
											**iCS**			**TAU**			**iCS vs. TAU**		
	**T0 Mean (SD)**	**T1 Mean (SD)**	**T2 Mean (SD)**	**T0 Mean (SD)**	**T1 Mean (SD)**	**T2 Mean (SD)**	***df***	***F***	***p***	η_p^2	**T0 vs. T1**	**T0 vs. T2**	**T1 vs. T2**	**T0 vs. T1**	**T0 vs. T2**	**T1 vs. T2**	**T0**	**T1**	**T1**
IR free[a]	7.00 (4.88)	11.81 (7.66)	10.97 (7.84)	6.96 (5.80)	9.75 (6.31)	9.42 (6.55)	1.60, 84.93	1.52	0.226	0.028									
IR cued	16.39 (8.61)	16.84 (8.49)	19.29 (10.14)	13.21 (7.81)	13.75 (8.17)	15.08 (7.58)	2, 106	0.30	0.745	0.006									
DR free[a]	1.74 (1.67)	4.03 (3.58)	3.71 (3.58)	1.71 (2.49)	1.87 (2.49)	2.25 (2.44)	1.74, 92.33	4.83	**0.013**	0.084	**< 0.001**	**< 0.001**	1.00	1.00	0.999	1.00	0.953	**0.015**	0.093
DR cued[a]	6.06 (3.71)	5.42 (3.63)	5.94 (3.84)	5.29 (3.38)	5.25 (4.10)	5.33 (3.68)	1.54, 81.50	0.28	0.701	0.005									

Note: Results of pairwise comparisons (Bonferroni correction) for T0, T1, and T2 assessments (iCS vs TAU). Results of pairwise comparisons (Bonferroni correction) for iCS and TAU groups (T0 vs T1, T0 vs T2, and T1 vs T2). The information in bold is statistically significant at an alpha level of 5%.

Abbreviations*:* FCSRT IR = Free and Cued Selective Reminding Test Immediate Recall; FCSRT DR = Free and Cued Selective Reminding Test Delayed Recall; iCS = individual Cognitive Stimulation; TAU = Treatment as usual; T0 = Baseline assessment; T1 = post-intervention assessment; T2 = 12-week follow-up assessment.

[a] Greenhouse–Geisser correction.

SUPPLEMENTARY INFORMATION

Table S5. FAB subtests. Results of repeated measures ANOVA.

	iCS (n = 31)			TAU (n = 24)			Group × time				Pairwise comparisons								
											iCS			TAU			iCS vs. TAU		
	T0 Mean (SD)	T1 Mean (SD)	T2 Mean (SD)	T0 Mean (SD)	T1 Mean (SD)	T2 Mean (SD)	*df*	*F*	*p*	η_p^2	T0 vs. T1	T0 vs. T2	T1 vs. T2	T0 vs. T1	T0 vs. T2	T1 vs. T2	T0	T1	T1
FAB conceptualization[a]	1.16 (0.78)	1.58 (0.99)	1.52 (1.09)	1.25 (0.99)	1.25 (0.90)	1.33 (0.92)	1.77, 93.67	1.45	0.240	0.027									
FAB mental flexibility	1.58 (0.89)	1.58 (1.13)	1.52 (0.85)	1.29 (0.75)	1.13 (0.80)	1.13 (0.90)	2, 106	0.26	0.774	0.005									
FAB motor programming[a]	1.45 (0.96)	1.58 (0.92)	1.52 (0.96)	1.42 (0.93)	1.50 (0.98)	1.46 (0.93)	1.77, 93.90	0.02	0.977	0.000									
FAB sensitivity to interference	1.10 (1.19)	1.52 (1.18)	1.35 (1.20)	1.54 (1.22)	1.33 (1.24)	1.17 (1.40)	2, 106	2.61	0.078	0.047									
FAB inhibitory control[a]	0.97 (1.05)	1.23 (1.23)	1.29 (1.32)	1.21 (1.14)	1.08 (1.21)	1.13 (1.12)	1.62, 85.62	1.22	0.295	0.022									
FAB environmental autonomy[a]	2.65 (0.61)	2.77 (0.43)	2.74 (0.44)	2.63 (0.65)	2.33 (0.87)	2.50 (0.72)	1.70, 89.99	3.28	**0.050**	0.058	0.941	1.00	1.00	0.144	0.946	0.288	0.906	**0.017**	0.132

Note: Results of pairwise comparisons (Bonferroni correction) for T0, T1, and T2 assessments (iCS vs TAU). Results of pairwise comparisons (Bonferroni correction) for iCS and TAU groups (T0 vs T1, T0 vs T2, and T1 vs T2). The information in bold is statistically significant at an alpha level of 5%.

Abbreviations: FAB = Frontal Assessment Battery; iCS = individual Cognitive Stimulation; TAU = Treatment as usual; T0 = Baseline assessment; T1 = post-intervention assessment; T2 = 12-week follow-up assessment.

[a] Greenhouse–Geisser correction.
